# Biopharmaceutical Profiling of New Antitumor Pyrazole Derivatives

**DOI:** 10.3390/molecules191016381

**Published:** 2014-10-13

**Authors:** Valentina Anuta, George Mihai Nitulescu, Cristina Elena Dinu-Pîrvu, Octavian Tudorel Olaru

**Affiliations:** Faculty of Pharmacy, Carol Davila University of Medicine and Pharmacy, Traian Vuia 6, Bucharest 020956, Romania; E-Mails: vali_anuta@yahoo.com (V.A.); ecristinaparvu@yahoo.com (C.E.D.-P.); octav_olaru2002@yahoo.com (O.T.O)

**Keywords:** acylthiourea, pyrazole derivatives, solubility, FaSSIF, FeSSIF, ADME prediction

## Abstract

Several new pyrazole derivatives have demonstrated promising antiproliferative and cytotoxic effects, but their poor solubility raised concerns over possible biopharmaceutical limitations. In order to improve their pharmaceutical potential we performed the biopharmaceutical profiling for nine pyrazole compounds using *in vitro* and computational methods. The experimental solubility was determined in five different media using a validated HPLC method. Although the experimental solubility was lower than the predicted one, a good linear relationship was observed. The results also indicated a minimal impact of endogenous tensioactives on solubility, suggesting dissolution rate limited absorption. The *in silico* experiments were focused on identification of molecular determinants of solubility, evaluation of drug-likeness, prediction of *in vivo* absorption based on mechanistic models, as well as identification of the main factors that could impact on the oral bioavailability. The results suggested that dose, solubility and particle size are the main determinants of absorption, whereas permeability has little effect, confirming the BCS Class II behavior of the compounds. The present investigation was able to rank the tested compounds in terms of biopharmaceutical behavior, and indicated the B3 series compounds as having a more favorable absorption profile making them the main candidates for advance to the pre-clinical *in vivo* studies.

## 1. Introduction

Acylthiourea derivatives represent an important focus of the synthetic efforts of many scientific groups in the pharmaceutical field. 1-Aroyl-3-substitued thioureas can be easily obtained, are versatile starting materials for the synthesis of a wide variety of other compounds and have a large spectrum of biological effects [1]. A series of 1-acyl-3-(2'-aminophenyl)-thiourea derivatives were synthesized and their anti-intestinal nematode activities were evaluated [2]. New benzoylthiourea derivatives showed potent cytotoxic properties toward pathogenic *Acanthamoeba* parasites [3]. Several studies have presented new acylthioureas and their antimicrobial effects. A series of 1-(isomeric fluorobenzoyl)-3-(isomeric fluorophenyl)thioureas were synthesized and showed good *in vitro* antibacterial activity on *Bacillus subtili*s and *Pseudomonas aureginosa* strains [4]. Various urea, thiourea and acylthiourea derivatives containing (*R*)-2-amino-1-butanol were evaluated against *Mycobacterium tuberculosis* stains H37Rv and strain 43, with acylthiourea derivatives showing the best anti-tuberculosis effect [5]. Some 2-(phenoxymethyl)benzoyl thiourea derivatives showed promising antibacterial properties [6]. Several pyrazole-4-carbonyl thiourea derivatives demonstrated good antibacterial effects, especially on *Klebsiella pneumonia* [7,8]. Related 1*H*-pyrazole-4-carbonyl thiourea derivatives displayed good antifungal activities against *Gibberella zeae*, *Fusarium oxysporum* and *Cytospora mandshurica* [9].

The pyrazolecarbonyl thiourea moiety was explored also in the synthesis of antitumor derivatives. A series of 4-benzoyl-1,5-diphenyl-1*H*-pyrazole-3-carbonyl thiourea derivatives showed important antitumor effect and are suggested as potent candidates for leukemia, liver and colon cancer treatment [10]. Related compounds, derivatives of 1,3-diphenyl-1*H*-pyrazole-4-carboxylic acid, were synthesized and demonstrated antiproliferative effects by inhibiting a range of cyclin-dependent kinases [11].

**Figure 1:**
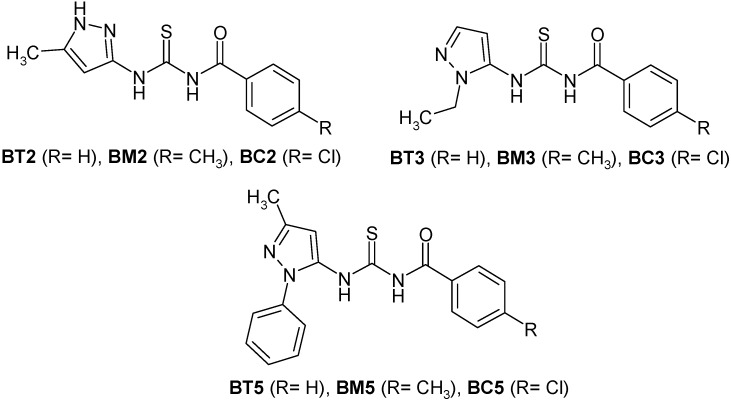
Chemical structure of the selected pyrazole derivatives.

In our previous studies [12,13] we synthesized various pyrazolecarbonyl thiourea and pyrazolyl thiourea derivatives, and tested them against different types of human cancer cells in order to evaluate their antiproliferative and cytotoxic effects. The most promising compound, *N*-benzoyl-*N*'-[3-(4-bromophenyl)-1*H*-pyrazol-5-yl]-thiourea presented logCI_50_ values under −4 in 43 of the NCI 60 cancer lines, values ranging from −4.12 to −5.75, with an average of −5.02. The best results were recorded on ovarian and the renal cancer cells [12]. Thus, further studies were focused on synthesis and cytotoxic evaluation of new derivatives containing aminopyrazole, thiourea and benzoyl moieties [13]. Most of the synthetized compounds presented cytotoxic effect similar to the reference substance, (colchicine) in the *Triticum aestivum* embryonic root length test, correlated with a low general toxicity [13]. Based on the results from the cytotoxicity assay, nine different compounds, belonging to three different analogue series (Figure 1) were selected for further evaluation. The compounds were selected to offer the best structural diversity. Their biopharmaceutical profiling represents the aim of present paper.

Although the preliminary cytotoxicity results suggested comparable activity for all the selected compounds, their poor solubility exhibited during the *in vitro* experiments raised concerns over their possible biopharmaceutical limitations.

Discovery and development of new drugs is a very lengthy and costly process, with extremely high failure rates [14]. A number of studies have already identified poor pharmacokinetic (PK) and ADME properties as being the most prominent cause of failure [15]. Therefore, a biopharmaceutical assessment of drug substances is crucial in order to evaluate their potential to display satisfactory properties and to rate candidate molecules in terms of their “drug-like” properties. As a consequence, weak drug candidates can be dropped in the early stages of drug development in order to focus the available resources to be most promising potential drug candidates.

Some systematic approaches to biopharmaceutical profiling were introduced based on Biopharmaceutics Classification System (BCS) [16], which provided a framework to consider key factors (dose, solubility, dissolution rate and permeability) that may influence *in vivo* performance of a drug product.

Since evaluation of solubility in aqueous buffer media is proved to have rather poor prediction power [17], dissolution profiling has evolved towards using physiologically relevant media [18,19,20,21,22] that better mimic the *in vivo* fluids from different segments of the gastrointestinal tract by taking into consideration the solubilizing effect of physiological surface active agents. More recently, the concepts of “drugability” [23] and “developability” [24] substantiated the development of molecular properties based criteria which are characteristic for compounds with appropriate biopharmaceutical profile [25]. Different computational and *in vitro* approaches were used to assess molecular properties correlated with oral absorption and bioavailability [26,27].

The growing interest in the biopharmaceutical characterization of both drug candidates and pharmaceutical products was reflected in the development of several *in silico* tools capable to forecast drug absorption and to identify critical factors influencing bioavailability based on different mechanistic absorption models *i.e.*, GastroPlus^TM^ which is based on the advanced compartmental absorption and transit ACAT model, Simcyp^TM^, which is based on the advanced dissolution absorption and metabolism (ADAM) model or PK-SIM^TM^ [28,29,30,31,32].

Since the pyrazolyl thiourea derivatives taken into the present study are poorly soluble compounds, their biopharmaceutical profiling arises as a necessity and the current paper presents their screening. The obtained profile was used to rank the selected compounds in terms of developability. This research also suggests a pharmaceutical risk assessment strategy that goes beyond the usual characterization of a clinical candidate molecule, in order to prioritize candidates for advance to the pre-clinical *in vivo* studies.

## 2. Results and Discussion

### 2.1. Preliminary Screening

#### 2.1.1. Molecular Descriptors

**Figure 2:**
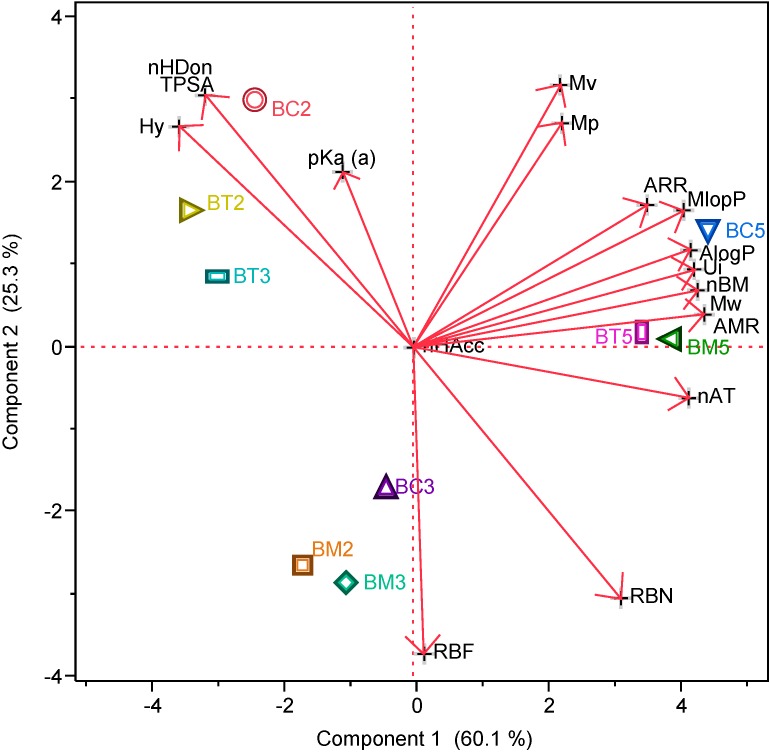
Principal Components Analysis (PCA) showing the position of the tested compounds according to their selected molecular descriptors.

The values for the calculated descriptors are presented in Table 1, whereas the PCA plots show how the principal components absorb the variation in the data (Figure 2).

**Table 1:** Values for the selected molecular descriptors.

Comp.	MW	pKa(a)	nHDon	nHAcc	nBM	RBN	nAT	TPSA	MlogP	AlogP	AMR	Ui	Hy	Mv	ARR	Mp
**BC2**	294.8	9.85	3	4	13	2	30	101.9	2.59	3.09	79.2	3.81	1.25	0.69	0.55	0.73
**BC3**	308.8	8.5	2	4	13	3	33	91.0	2.45	3.08	82.9	3.81	0.46	0.67	0.52	0.72
**BC5**	370.9	8.84	2	4	19	3	40	91.0	3.7	4.59	102.6	4.32	0.31	0.7	0.63	0.74
**BM2**	274.4	8.91	2	4	13	3	33	91.0	1.91	2.42	78.1	3.81	0.45	0.65	0.55	0.69
**BM3**	288.4	8.05	2	4	13	3	36	91.0	2.18	2.9	83.2	3.81	0.41	0.64	0.52	0.69
**BM5**	350.5	8.25	2	4	19	3	43	91.0	3.44	4.41	102.8	4.32	0.27	0.67	0.63	0.71
**BT2**	260.4	8.8	3	4	13	2	30	101.9	2.05	2.42	74.4	3.81	1.25	0.67	0.58	0.71
**BT3**	274.4	7.91	3	4	13	2	33	101.9	2.32	2.91	79.4	3.81	1.2	0.65	0.55	0.69
**BT5**	336.5	8.1	2	4	19	3	40	91.0	3.20	3.93	97.8	4.32	0.3	0.68	0.65	0.72

#### 2.1.2. Drug-Like Indices

Special emphasis was put on evaluation of the drug-like indices, since they can act as useful filters to identify good drug candidates from large collections of compounds. The “drug-like” indices calculated by E-Dragon are dummy variables taking value equal to 1 when all the criteria of the consensus definition of a drug-like molecule are satisfied and 0 otherwise. Two different classes of indices were evaluated:

(a) A biopharmaceutical predictor -Lipinski Alert Index (LAI)- based on the “Lipinski Rule of 5” [33], which predicts that poor absorption or permeation occurs if nHDon > 5, nHAcc > 10, MW > 500 and MlogP > 4.15.

(b) A group of eight drug-like indices were proposed by Ghose-Viswanadhan-Wendoloski [34], derived from analysis of the distribution of some physicochemical properties (AlogP, AMR, MW, nAT) and chemical constitutions of drug molecules available in the Comprehensive Medicinal Chemistry (CMC) database. Ghose-Viswanadhan-Wendoloski drug-like index (GVWI) provides a global evaluation of “drug likeliness” whereas the rest of seven indices are dedicated to different classes of drugs *i.e.*, antiinflammatory-like index (Inflammat), antidepressant-like index (Depressant), antipsychotic-like index (Psychotic), antihypertensive-like index (Hypertens), hypnotic-like index (Hypnotic), antineoplastic-like index (Neoplasic) and antiinfective-like index (Infective), were evaluated. Two different ranges have been proposed: the qualifying range covering approximately 80% of the active studied drugs and the preferred range defined as the smallest range within the qualifying range containing approximately 50% of the drugs [34].

For the particular case of the Neoplasic-50 index, a compound with antineoplasic activity has AlogP between 0.0 and 3.7, AMR is between 60 and 107, MW between 258 and 388, the total number of atoms 30 to 55, and contains at least one of the structural characteristics mentioned above. The values obtained for the evaluated indices as well as values of the molecular descriptors used for their assessment are presented in Table 2.

**Table 2:** Assessment of drug-like indices for the selected compounds.

*Compound*	*LAI*	*GVWI* *	*Inflammat*	*Depressant*	*Psychotic*	*Hpertens*	*Hypnotic*	*Neoplasic*	*Infective*
**BC2**	0	1	1	0	0	0	0	1	1
**BC3**	0	1	1	0	0	0	0	1	1
**BC5**	0	0	0	0	0	0	0	0	0
**BM2**	0	1	0	0	0	0	0	1	1
**BM3**	0	1	1	0	0	1	0	1	1
**BM5**	0	0	0	0	0	0	0	0	0
**BT2**	0	1	0	0	0	0	0	1	1
**BT3**	0	1	1	0	0	0	0	1	1
**BT5**	0	1	0	0	0	0	0	0	0

Note: * evaluated for the 50% range.

Since LAI is an “alert index” a 0 value suggests that none of the poor absorption or permeation criteria are met, therefore all the compounds are likely to have an adequate biopharmaceutical profile.

In terms of drug class likeliness, the indices based on the more restrictive preferred range (50%) were used. The compounds don’t seem to be active on the Central Nervous System, whereas **BC5**, **BM5** and **BT5** are not drug-like structures at all in terms of GVWI. The other compounds have structure and molecular properties favorable for both antineoplastic and antiinfective activity.

### 2.2. Quantitative HPLC Analysis

The HPLC method employed method for the pyrazole derivatives quantitative analysis was found to be linear in the range 0.1–10 μg/mL, with a mean with a mean R^2^ value >0.999 for all the tested compounds (Table 3). The limit of quantification (LOQ), calculated based on a 10:1 signal-to-noise ratio was of about 0.02 µg/mL (ranging from 0.016 µg/mL for **BM2** to 0.024 µg/mL for **BT3**).

**Table 3:** Linear regression data for the calibration curve (n = 3).

Compound	Slope ± SD	Intercept ± SD	R^2^
**BC2**	45090 ± 602.1	7630 ± 2620	0.9991
**BC3**	42700 ± 559.5	−930.1 ± 2435	0.9991
**BC5**	46360 ± 191.8	2016 ± 834.7	0.9999
**BM2**	52610 ± 350.9	915.1 ± 1527	0.9998
**BM3**	49990 ± 435.1	1287 ± 1894	0.9996
**BM5**	48000 ± 676.5	−2474 ± 2944	0.9990
**BT2**	38650 ± 526.9	5702 ± 2293	0.9991
**BT3**	36090 ± 243.6	5524 ± 1051	0.9998
**BT5**	45620 ± 381.8	4301 ± 1647	0.9996

**Figure 3:**
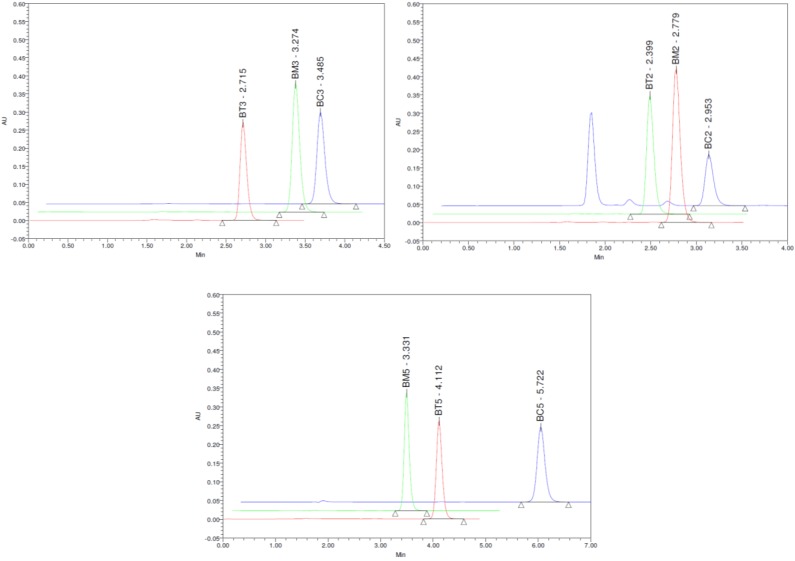
Representative chromatograms of standard samples containing 10 µg/mL of each tested compound.

Repeatability and intermediate reproducibility experiments resulted in RSD values ranging from 0.24% to 4.2%. The bias (%) between the experimental and nominal concentrations of the quality control samples did not exceed 4.5%. All these results indicated that precision and accuracy of the assay are satisfactory. Representative chromatograms obtained for the analyzed pyrazole derivatives depicted in Figure 3.

### 2.3. Equilibrium Solubility Study

Solubility is a crucial parameter for successful drug development, as poor solubility could compromise the pharmacokinetic and pharmacodynamic profile of drug candidate molecules [35,36].

The equilibrium solubility experiments for the selected pyrazole derivatives were performed in five media simulating different segments of the GI tract (three aqueous solutions—SGF, SIF and pH 4.5 acetate buffer) and two biorelevant media containing physiological surface active agents (FaSSIF and FeSSIF).

**Figure 4:**
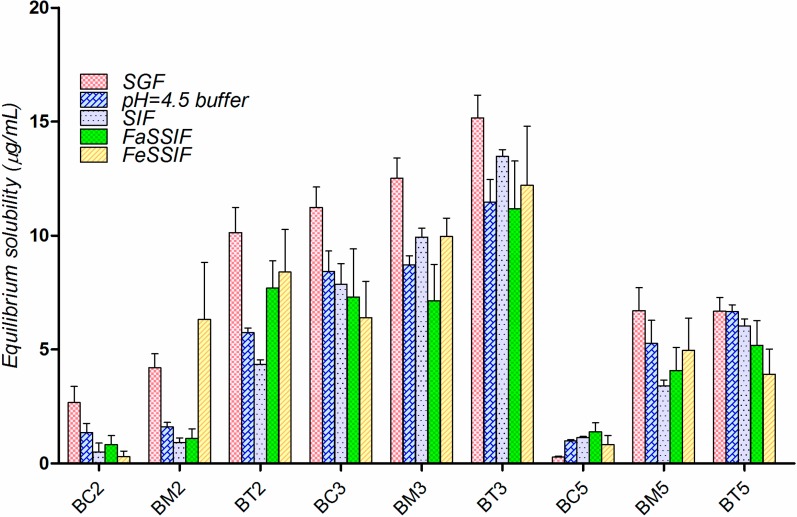
Solubility of the tested compounds in simulated gastro-intestinal fluids (μg/mL), at 25 °C (mean for three determinations).

The experimental solubility of the tested compounds in the above media is presented in Figure 4. The overall solubility hierarchy for the tested compounds is **BT3** > **BM3** > **BC3** > **BT2** > **BT5** > **BM5** > **BM2** > **BC5** ≈ **BC2**. Solubility appeared to be slightly higher in the simulated gastric fluid (SGF, pH = 1.2), and decreased as the pH increased.

Solubility in biorelevant media is generally higher compared to solubility measured in corresponding aqueous buffers [37,38] due to the wetting and/or solubilizing effect of micelles formed from sodium taurocholate and lecithin from FaSSIF and FeSSIF composition [39,40]. However, experimental data revealed very limited effect of the physiological surfactants on the solubility of tested compounds.

In order to explain this somewhat “strange” behavior we have to take into account the fact that aqueous solubility (logS_w_) of any organic non-electrolyte or weak electrolyte was found to be directly correlated with two molecular properties: octanol-water partition coefficient (logP) and melting point (Mp, °C ) of the compound [41,42,43]. The general solubility equation (GSE) developed by Jain *et al.* [43] provides a quantitative relationship between the three parameters:

log *S_w_* = 0.5 − 0.01(*Mp* − 25) − log *P* (1)

**Figure 5:**
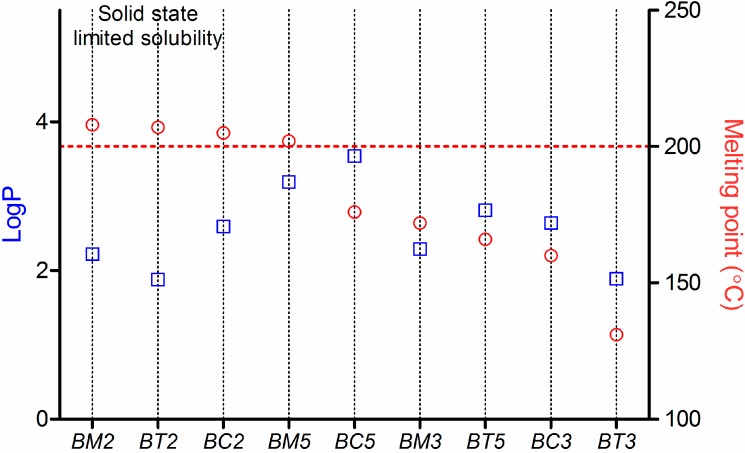
Analysis of the tested compounds from the general solubility equation (GSE) point of view.

For the tested compounds, the logP and Mp values are plotted in Figure 5.

Starting from the general solubility equation, the poorly soluble drugs were sub-classified by the group of Bergström *et al.* [44] in two types of molecules: “grease ball” and “brick dust” molecules. The “grease ball” compounds are highly lipophilic (logP > 4) with a low melting point (<190–200 °C). These compounds cannot form bonds with water molecules, and therefore their solubility is limited by the solvation process. By contrast, “brick dust” molecules are usually compounds with rather rigid and flat molecules with a high melting point (>190 °C) and moderate lipophilicity. Their solubility is being solid-state-limited being mainly restricted by the inability of the compound to dissociate from the crystal lattice and can generally be improved by different methods involving a disruption of the stable crystal lattice [45,46].

In the particular case of the tested compounds (Figure 5), **BM2**, **BT2**, **BC2** and **BM5** have tipical “brick dust”-type properties: their Mp is >200 °C, and lipophilicity expressed as logP ranges between 1.8 and 3.2. The other compounds have a intermediar behaviour, but their solubility is likely to be influenced in a greater extent by their crystal structure than by solvation. As a consequnce, the influence of the surface active agents on their solubility is minimal.

Moreover, in case of four of the nine tested compounds, solubility in FaSSIF decreased compared to SIF solubility. Electrostatic interactions between the compounds and the bio-relevant medium components (lecithin and taurocholic acid) could provide an explanation for that behavior.

At pH 6.5, the theoretical distribution of the ionic species for the analyzed pyrazole derivatives suggested about 80% contribution of the neutral and 20% contribution of the negatively charged form.

As lecithin is zwitterionic and taurocholic acid is fully deprotonated at pH 6.5, FaSSIF contains a net negative charge, and this could explain the different behavior observed for acids and bases, it is very likely that the negative charge of the compounds is detrimental for their solubility because of electrostatic repulsion with the media components [40].

**Figure 6:**
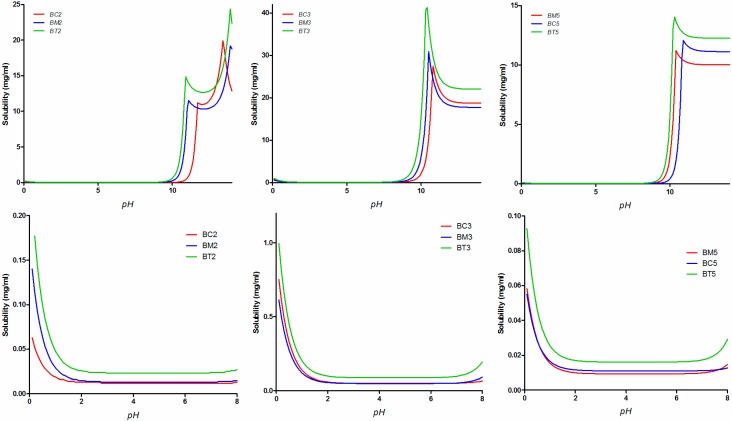
Simulated solubility *vs.* pH profiles for the tested compounds over the whole pH range (**top** row) and only in the physiologically relevant range (**bottom** row).

The experimental solubility results were compared with the *in silico* predictions of solubility performed with the GastroPlus^TM^ software (Figure 6).

**Figure 7:**
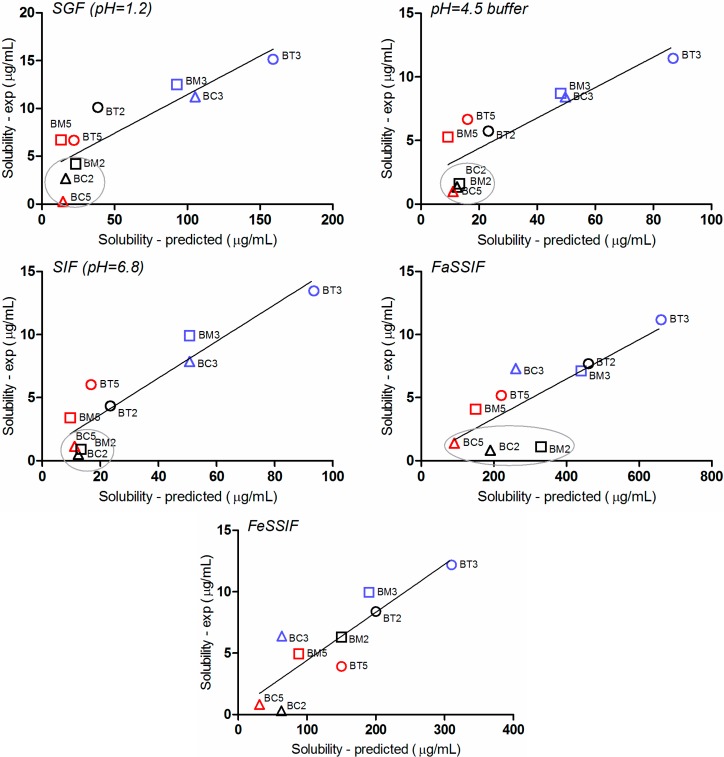
Correlation between the simulated and experimental solubility values in the five tested media.

The correlation between the *in silico* predicted and experimental solubility in the five tested media is presented in Figure 7. Although the experimental solubility is lower than the predicted one, a good linear relationship was generally observed.

The FaSSIF media generated the highest discrepancies between the estimated and *in vitro* experimental values. This is somewhat to be expected, since bile salts concentration in the medium (3 mM) is in the range reported in literature to correspond to sodium taurocholate critical micellar concentration (CMC) (1–12 mM) [47], and in many cases, at concentrations in the proximity of CMC a quite “erratic” behavior can occur [48].

Two other important aspects can be observed. Firstly, the simulation software predicts an important contribution of solvation on solubility (and therefore a major impact of physiological surfactants), whereas the experimental results prove a solid-state limited solubility behavior. As a result, solubility in biorelevant media is significantly overestimated by the software.

Secondly, for three of the compounds (**BM2**, **BC2** and **BC5**) the solubility is systematically underestimated in all dissolution media. It is to note that **BM2** and **BC2** have typical “brick dust” characteristics (Figure 5), whereas for each of the three base structures of the tested compounds, the chlorinated molecule presented the lowest solubility.

### 2.4. Impact of the Molecular Descriptors on Solubility in Different Media

**Figure 8:**
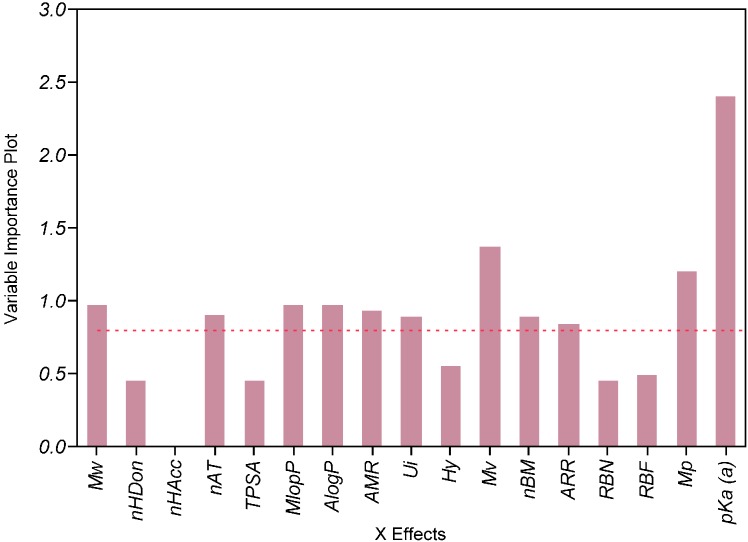
Evaluation of importance of calculated predictors on experimental solubility in FaSSIF by PLS analysis.

The PLS analysis suggested that whereas ionization was the most influential factor, other descriptors related to lipophilicity (MlogP, AlogP), size and polarizability (MW, nAT, Mv, Mp, AMR) had a significantly negative impact on solubility (Figure 8). The results also suggested a significant impact of the aromaticity degree pointing therefore towards solid-state limited solubility [49,50] of the compounds.

### 2.5. Biopharmaceutical in Silico Profiling

A selective presentation of the main physicochemical and pharmacokinetic parameters evaluated *in silico* is presented in Table 4.

**Table 4:** The main biopharmaceutical descriptors of the tested compounds, as simulated by GastroPlus^TM^.

Compound	logD	Ref pH ^a^	Aq Sol ^b^ (µg/mL)	Diff Coef (cm^2^/s*10^5^)	Peff (cm/s*10^4^)	fp (%)	RBP	MAD (mg)	%Fa ^c^
**BC2**	2.59	6.9	12.4	0.81	2.03	19.9	0.82	112.2	55.4
**BC3**	2.64	6.14	49.9	0.78	3.29	16.03	0.78	744.9	99.6
**BC5**	3.54	6.64	11.1	0.69	3.99	6.68	0.71	244.7	79.9
**BM2**	2.22	6.58	13.3	0.8	1.79	24.68	0.88	101.6	42.9
**BM3**	2.29	5.92	48.4	0.77	2.88	17.73	0.78	618.6	99.5
**BM5**	3.19	6.4	9.4	0.69	3.47	7.3	0.75	165.6	72.4
**BT2**	1.88	6.41	23.3	0.84	1.64	25.48	0.94	158.7	81.2
**BT3**	1.89	5.71	87.3	0.8	2.56	17.05	0.82	972.8	99.6
**BT5**	2.81	6.2	16.2	0.71	3.03	6.9	0.78	231.9	85.5

Notes: ^a^ Ref pH (“native pH”)—pH of the compound-saturated water solution; ^b^ estimated at Ref pH; ^c^ evaluated for a suspension containing 100 mg tested compound, with a mean particle size of 25 µm.

It is noteworthy that for all analyzed molecules the predicted effective human jejunal permeability values are higher than Peff of metoprolol, which is generally accepted as borderline between low- and high-permeability behavior [51,52], therefore they are to be classified as high permeability compounds. In conjunction with their low solubility behavior, the results indicate that they are to be classified as BCS Class II compounds [16].

The MAD values are significantly higher for **BC3**, **BM3** and **BT3** (2.5 up to 9 times higher than the rest), as they also are the only compounds with complete absorption under the simulation conditions (suspension containing 100 mg tested compound, with a mean particle size of 25 µm).

**Figure 9:**
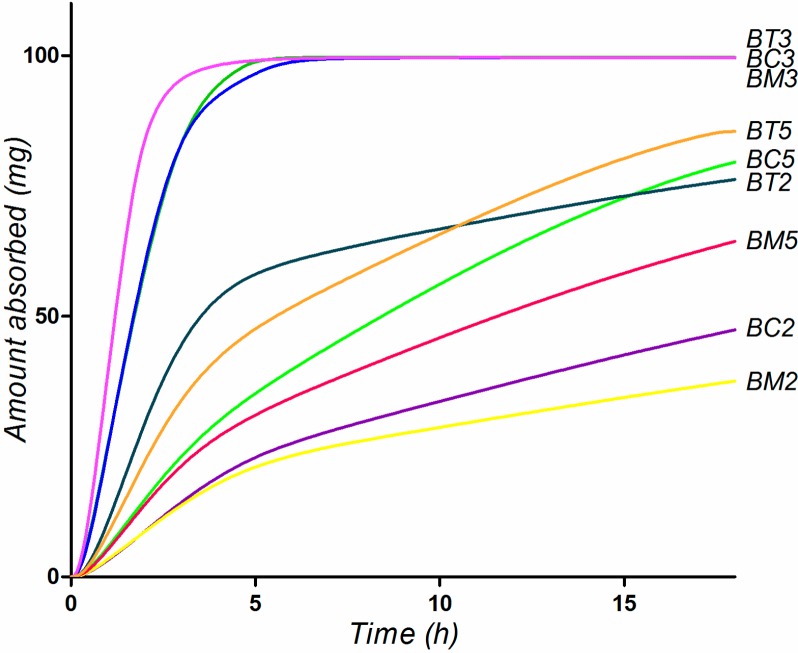
Simulated absorption profiles for the tested compounds, considering oral administration of 100 mg compounds as aqueous suspension.

Simulation of oral absorption performed in the Single Simulation Mode of GastroPlus^TM^ the Human-Physiological-Fasted physiological model with the default Opt logD SA/V 6.1 Absorption Scale Factor (ASF) parameters resulted in the simulated absorption profiles presented in Figure 9. Since the experimental data suggested no significant effect of the physiological surfactants, no bile salts solubilizing effect was considered in the simulations. The simulation predicts a rapid and complete absorption of **BC3**, **BM3** and **BT3**, for the rest of the compounds absorption being slow and incomplete (Figure 9).

**Figure 10:**
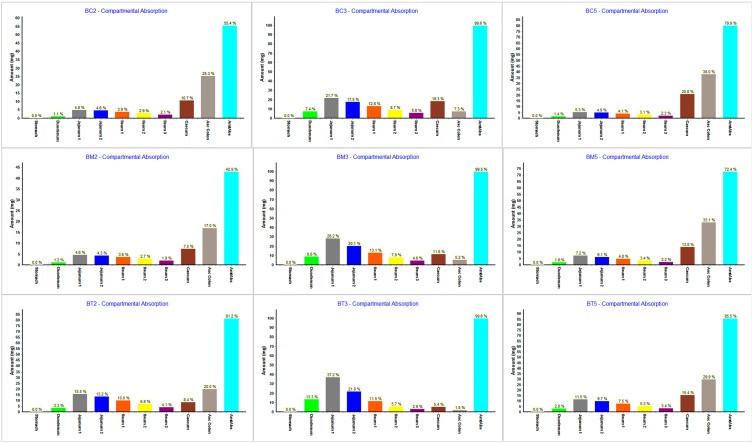
Gastrointestinal simulation of regional absorption distribution for the tested compounds.

The regional absorption distribution for the tested compounds obtained from the simulations using ACAT model provided some important information (Figure 10).

As expected, for the higher solubility compounds (**BC3**, **BM3** and **BT3**) the simulations suggested that absorption will occur almost completely in the small intestine. This is in accordance with the fact that small intestine represents the optimal absorption site.

Within the different intestinal compartment, the absorbed fraction decreases from the jejunum 1 to ileum 3 probably due to shorter transit time along the different segments. For the other compounds, simulations suggested a important contribution of the ascending colon in drug absorption, since this segment has a high residence time and sufficient available free water volume to allow absorption [53].

**Figure 11:**
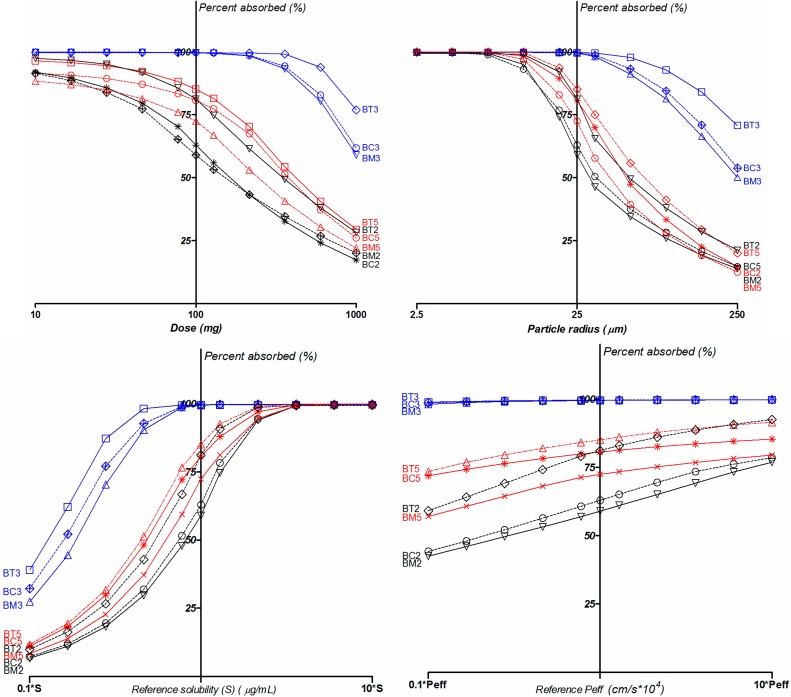
PSA analysis for dose, solubility at reference pH, particle radius and effective human jejunal permeability (Peff).

Parameter sensitivity analysis (PSA) showed the influences of the various input variables on the absorbed fraction (Figure 11). The results suggested that a smaller dose or increased solubility would improve both bioavailability and the percentage of absorption, whereas, in comparison, an increase in permeability would have little effect, confirming the BCS Class II behavior of the compounds.

In terms of the effective particle radius, a variation from 2.5 μm to 250 μm was considered in the simulation. Oral exposure was found to be very sensitive to the particle size for the B5 and B2 series, and much more “inert” for B3 series compounds.

Nearly complete oral absorption (*i.e.*, Fa > 85%) could be achieved with particle radius up to about 10 μm for 2 and 5 series, 90 µm for **BC3** and **BM3** and up to 150 µm for **BT3**. Under this condition, particle size reduction can be an effective method to improve the dissolution rate, and therefore increase bioavailability.

Meanwhile, PSA also showed that the dose highly influences the absorption rate. A range of 10–1000 mg was screened for in the simulation. Only doses up to 17 mg for **BM5**, **BC2** and **BM2**, 50 mg for **BC5**, 77 mg for **BT2** and **BT5**, and 360 mg for **BC3**, **BM3** and **BT3** lead to >85% compound absorbed.

Variations in solubility is a natural consequence of intraindividual variability, but also can be altered in the formulation process (for instance amorphization, lipid or nanoscaled formulations), therefore its impact on bioavailability is of the utmost importance. The PSA on solubility impact on absorption provided results consistent with the other simulations: small changes in solubility led to significant bioavailability variations for the 2 and 5 series, whereas for the 3 series absorption was less sensitive to solubility variations.

## 3. Experimental Section

### 3.1. Chemicals

Physiological compounds—granular lecithin (Acros Organics, Geel, Belgium) and sodium taurocholate 97% (Sigma, St. Louis, MO, USA) were used. HPLC grade acetonitrile and methanol was purchased from Merck KGaA (Darmstadt, Germany). Trifluoroacetic acid for HPLC was purchased from Sigma-Aldrich. Water for chromatography (resistivity minimum 18.2 MΩ and TOC maximum 30 ppb) was produced within the laboratory by means of a TKA GenPure system and used during the experiments. The pyrazole compounds were synthesized according to the procedures described in previous research [12,13]. All other reagents were of analytical grade, purchased from different commercial suppliers and used without further purification.

### 3.2. Quantitative HPLC Analysis

The quantitative analysis of the selected compounds was carried out using a Waters liquid chromatographic system (Waters, Milford, MA, USA) consisting of consisting of a 600 E Multisolvent Delivery System, Waters AF in line degasser, 486 UV tunable absorbance detector and Waters 717 plus autosampler. Empower Pro software (Waters) was used to control the instrument, acquire and process data. The chromatographic separation was achieved on a Hypersil Gold, 5-μm 150 × 4 mm column (Thermo Fisher Scientific, Waltham, MA, USA) maintained under constant temperature (30 °C). The mobile phase consisted of an isocratic mixture of 0.1% trifluoroacetic acid-acetonitrile (50:50 v/v), delivered at 1.0 mL/min flow rate. The detector was set at the wavelength corresponding to the maximum absorbance of each compound in UV spectra.

The HPLC method was subjected to validation in accordance with the International Conference on Harmonization (ICH) regulations Q2(R1) [54] in terms of specificity, linearity, precision (repeatability and intermediate precision) and accuracy.

Assay specificity was examined in relation to interference from matrix components in the drug-free media used for determination of solubility.

The linearity assessment, designed to measure the capability of the method to produce results related in a linear way to the concentrations of the analytes, was performed for each compound by using seven concentration levels, in the range 0.1–10 µg/mL. All analyses were performed in triplicate. Calibration curves and corresponding determination coefficients (R^2^) were calculated by least squares linear regression analysis.

For each compound, detection limit (LOD) and quantitation limit (LOQ) were determined based on the signal-to-noise ratio. The concentrations yielding to signal-to-noise ratios of 3:1 and 10:1 were taken as LOD and LOQ, respectively.

Precision was evaluated for repeatability and intermediate reproducibility on spiked samples, at three different concentration levels (QC_low_, QC_medium_ and QC_high_). Precision was assessed by means of RSD% values computed for absolute peak areas resulting from interpolation on the corresponding calibration curves. Repeatability study was achieved by injection of five replicates from a single prepared spiked plasma sample within a single day experimental session, whereas intermediate reproducibility was tested by means of five different samples processed in different experimental sessions for each concentration level. The bias (%) between the concentration values determined for the QC samples and their nominal values was used as accuracy indicator.

### 3.3. Evaluation of Experimental Thermodynamic Solubility

Thermodynamic (equilibrium) solubility experiments were performed by using the saturation shake-flask method. Since our study was focused on oral administration route, solubility was tested in different media simulating gastric and intestinal conditions of the fasted and fed state. Hence, the USP Simulated Gastric Fluid (SIF, pH = 1.2), Simulated Intestinal Fluid (SIF, pH = 6.8) and 0.05 M acetate buffer pH = 4.5 were used in the experiments [55].

However, the *in vivo* solubility in the GI tract can be significantly higher than expected from *in vitro* solubility tests performed in buffer solutions due to presence in the intestinal lumen of natural lipids and bile salts with surface-active properties [19]. Therefore, the solubility profiling also included biorelevant dissolution media simulating the intestinal content *i.e.*, Fasted State Simulated Intestinal Fluid (FaSSIF) containing 3 mM sodium taurocholate at a pH of 6.5 and Fed State Simulated Intestinal Fluid (FeSSIF) with pH 5.0 and 15 mM sodium taurocholate concentration [18]. Preparation of FaSSIF and FeSSIF was performed using the method described recently by Jogia *et al.*, [56], without the use of methylene chloride.

For each experiment, excess of substance was carefully added to 1.5 mL of medium, in 2 mL Eppendorf polypropylene microtubes. The vials were capped, stirred for 30 s at 2500 rpm on an IKA Genius 3 vortex mixer (IKA Werke GmbH & Co. KG, Staufen, Germany) and maintained under mild agitation (250 rpm) for 24 h at 25 °C on a IKA HS 260 orbital shaker (IKA Werke).

The resulting samples were centrifuged at 12,000 rpm, for 10 min on a Hettich Mikro 220R centrifuge (Andreas Hettich GmbH & Co. KG, Tuttlingen, Germany). Aliquots of 0.25 mL were collected from the supernatant and diluted with mobile phase to a final volume of 1 mL. A volume of 10 µL from the resulting sample was injected into the HPLC system. All experiments were performed in triplicate.

### 3.4. In Silico Tools

More than 100 constitutional and topological descriptors, functional group counts, molecular properties were calculated by the E-Dragon Java based online platform [57,58]. However, only few of them have an actual impact on the biopharmaceutical properties of the selected compounds. Therefore, a Principal Components Analysis (PCA) based approach was further used in order to reduce dimensionality of the data set and facilitate interpretation. Starting from the matrix containing all variables the most significant descriptors were selected consulting the loading scores for the first three principal components. Based on this approach, the following descriptors were selected: molecular weight (MW), acid and base ionization constants (pKa and pKb), number of donor atoms for H-bonds (nHDon), number of acceptor atoms for H-bonds (nHAcc), number of multiple bonds (nBM), number of atoms (nAT), aromatic ratio (ARR), unsaturation index (Ui), hydrophilic factor (Hy) [59], Ghose-Crippen octanol-water partition coefficient (AlogP) [60], Moriguchi octanol-water partition coefficient (MlogP) [61], molar refractivity (AMR) [60], mean atomic polarizability (Mp), mean atomic van deer Waals volume (Mv) [59], number of rotatable bonds (RBN) [62], as well as topological polar surface area using N, O, S, P polar contributions (TPSA) [63].

The influence of the different descriptors on solubility was performed by means of multivariate data analysis applying partial least-squares projection to latent structures (PLS). The analysis of how molecular features are related to the the drug solubility in all selected media was performed using JMP^TM^, Version 11.2 (trial) software (SAS Institute Inc., Cary, NC, USA). The importance of descriptors was evaluated by the Variable Importance Plot (VIP), values which summarizes the importance of the x variables in the model. VIP values higher than 0.8 were considered.

Since the dataset was quite small, the obtained data were not regarded as predictive model, but were used as a tool to further investigate the properties of importance for poor solubility.

Further, a biopharmaceutical evaluation as to which physicochemical and formulation factors could have an impact on the oral bioavailability of the selected compounds was performed based on computer simulations performed with GastroPlus™ (version 8.0, Simulations-Plus Inc.; Lancaster, CA, USA) software.

The ADMET Predictor^TM^ module was used to generate estimates for many GastroPlus^TM^ inputs: MW (g/mol), molecular radius (Å), acid and base ionization constants, (pKa or pKb), octanol/ water partition coefficient (logP); distribution coefficient at physiological pH = 7.4 (logD) aqueous solubility (Aq Sol, mg/mL), solubility in biorelevant media (mg/mL), diffusion coefficient (Diff Coeff, cm^2^/s × 10^5^), effective human jejunal permeability (Peff, cm/s × 10^4^), as well as some pharmacokinetic parameters, such as percent unbound in plasma (fp, %), Blood/Plasma Ratio (RBP) and volume of distribution (V_c_, L/kg). Additionally, the software allowed calculation of Maximum Absorbable Dose (MAD, mg), an important parameter in assessment of drug “developability” [64].

The pKa information was further used to evaluate solubility *vs.* pH and logD *vs.* pH profiles (used in pH dependent dissolution and absorption models).

The Advanced Compartmental Absorption and Transit Model (ACAT) model [28] implemented in GastroPlus^TM^ was used to predict the rate and extent of oral absorption. Simulation of oral absorption was performed in the GastroPlus^TM^ Single Simulation Mode with the default Absorption Scale Factor (ASF) values in the Human-Physiological-Fasted mode. The ACAT model of the human gastrointestinal tract used for the simulations comprises nine successive compartments, the first one representing the stomach and subsequent compartments corresponding to duodenum, jejunum (two compartments), ileum (three compartments), cecum, and ascending colon.

Parameter sensitivity analysis (PSA) was performed in order to identify critical parameters for oral absorption. PSA was performed using the GastroPlusTM build-in PSA simulation mode, in order to identify the critical parameters for oral absorption. PSA was performed with range from one tenth to ten-fold the tested input values, *i.e.*, dose, reference solubility, particle radius as well as effective human jejunal permeability.

## 4. Conclusions

This paper evaluated the biopharmaceutical profile of several thiourea derivatives with antiproliferative and cytotoxic potential based on experimental equilibrium solubility studies and *in silico* prediction and modeling approaches. Based on the results from both *in silico* and *in vitro* experiments, the present investigation was able to rank the tested compounds in terms of biopharmaceutical behavior. The *in silico* simulations highlighted that only 1-ethyl-1*H*-pyrazol-5-yl derivatives (**BC3**, **BM3** and **BT3**) are completely absorbed from the GI tract and their absorption is not sensitive to the main critical formulation variables *i.e.*, dose and particle size, leading to low variability of their pharmacokinetic profile. The experimental *in vitro* studies also pointed out that the B3 series compounds are having the highest overall solubility in simulated gastric and intestinal media. Therefore, all results indicated that these compounds are having a more favorable absorption profile, making them the main candidates for advance to the pre-clinical *in vivo* studies. In addition, the results indicate that biopharmaceutical profiling represents a reliable method to improve the future prediction for similar compounds.
